# Application of an inducible transposon with anther culture in generation of di-haploid homologous mutants

**DOI:** 10.1186/1999-3110-55-27

**Published:** 2014-02-24

**Authors:** Hsiu-Chun Yang, Yuh-Chyang Charng

**Affiliations:** Department of Agronomy, National Taiwan University, No.1 Sec.4 Roosevelt Rd, Taipei, Republic of China Taiwan

**Keywords:** Anther culture, Rice, Inducible transposon, Knockout mutant, Di-haploid mutagenesis

## Abstract

**Background:**

Insertional mutagenesis represents one of the most effective ways to acquire information about a plant gene’s function. However, it is hindered by the autosomal genome being diploid and therefore, most mutations being recessive. The problem is addressed by inducing the transposition during anther culture so that selected mutations can be transmitted and then regenerated to a homozygous state.

**Results:**

To this end, we treated transgenic rice floral tissues containing the inducible transposon with an inducer, salicylic acid. Excision events were detected in regenerated calli and subsequent plantlets. DNA blot and PCR assay were used to determine the homogeneity of knockout mutants. About 5% of the mutants containing transposition events were homozygous. Furthermore, the inducible transposon was active during calli regeneration.

**Conclusions:**

This strategy could be applicable to improve transposition efficiency in microspore development stages to create stable di-haploid mutants in plants.

**Electronic supplementary material:**

The online version of this article (doi:10.1186/1999-3110-55-27) contains supplementary material, which is available to authorized users.

## Background

Transposable element (TE) tagging has become a powerful tool to create mutants for isolating new genes in animal and plant systems (Lin et al. 2006; Horie et al. 2011; Veilleux et al. 2012). Several experimental approaches have been used to develop transgenic plants with genes randomly tagged by insertion elements (Greco et al. 2001; Hirochika 2001; Hirochika et al. 2004; Izawa et al. 1997; Jeon and An 2001). One limitation of tagging experiments is the problem of the autosomal genome being diploid and thus, most mutations are recessive. Therefore, loss-of-function screens with mutant cells that lack expression of a particular gene are difficult in diploid cells, because in most cases both alleles of a gene must be knocked out to result in a phenotype. Homozygous lines are required for screening to obtain the desired mutant phenotype. Therefore, creation of biallelic TE-insertion mutants is time-consuming and requires more breeding processes.

For animal systems, insertional mutagenesis usually produces a heterozygous individual and then homozygous mutants by further intercrossing. An alternative technique has been used for phenotypic screening directly from somatic cells -- inducing loss of heterozygosity of the mutated gene, which is a less demanding technique with the application of high-concentration G418 selection in heterozygous cells (Huang et al. 2012). However, this technique has relatively low efficiency and sometimes creates a mutation with altered function in a non-targeted locus, with false results (George et al. 2007).

For plant systems, anther culture is used to produce haploids and di-haploids (DHs) via gametic embryogenesis for a single-step development of complete homozygous lines from heterozygous parents. Regeneration from male gametes has been reported in more than 200 species belonging to the Solanaceae, Cruciferae and Gramineae families (Dunwell 1986; Hu and Yang 1986). In rice, the regeneration rate is in general is more than 5 green plantlets/100 anthers. This technique has been further used with TE mutagenesis to obtain homozygous mutants (Kikuchi et al. 2003; Dong et al. 2012). TE allows for creating mutagenesis in abundant germline cells and the subsequent anther culture allows for producing DHs. Yet, these experiments have involved use of native TEs without attempting to promote transposition efficiency or controlling the transposition specifically in germinal cells. Thus, the transposition events may occur in regenerated somatic DH cells, which results in heterozygous mutant plantlets. Several successful transposon-tagging experiments in plants indicated that the *Ac*/*Ds* system is a valuable tool for rice functional genomic studies (Zhu et al. 2003,2004; Komatsu et al. 2003). Previously, we constructed a one-time inducible transposon, *COKC*, by fusing the transposase gene with a chemically inducible PR-1a promoter (Charng et al. 2008; Tai et al. 2011). *COKC* was introduced into rice plants and could be successfully induced by the inducer salicylic acid (SA) to trigger transposition events. Here, we assessed the use of TE mutagenesis with rice anther culture to produce biallelic mutants from *COKC*-containing transgenic rice.

## Methods

### Donor plants and pre-treatments of panicles

According to our previous experiments (Tai et al. 2011), we used five independent transgenic lines (K-17, K-19, K-20, K-21 and K-24) containing a single copy of *COKC* as anther donor plants. All transgenic rice plants were self-pollinated to obtain homozygous *COKC*. Transgenic rice seedlings were set in pods and grown in a greenhouse.

### Anther culture and SA induction

For controlled anther culture, the collected tillers were placed in a bottle containing water, covered with a polyethylene bag, and pretreated at 10°C for 10 days (Ogawa et al. 1995). Then, spikes with the leaf sheaths removed were surface-sterilized with 70% ethanol for 30 sec. Spikeles were removed from the sheath leaf, soaked in 0.6% NaOCl for 3 min, then rinsed thoroughly with sterile distilled water three times. Anthers at mid- to late-uninucleate microspore stages were inoculated onto callus induction medium (CIM) consisting of 1/2 MS salts except with full-strength Fe-EDTA and vitamins, supplemented with 4 mg NAA, 2 mg kinetin, 6% sucrose and 0.8% agar (Sigma Chemical Co., St. Louis, MO). In total, 50 anthers were plated in each Petri dish (87 × 15 mm) containing 25 ml solid medium. Dishes were sealed with paraffin taps (Whatman Ltd.) and maintained at 24°C for 7 to 9 weeks. Calli were transferred to plant regeneration medium, which consisted of MS salt and vitamins, supplemented with 1 mg/L NAA, 4 mg/L kinetin and 3% sucrose, and gelled with 0.3% phytagel. Calli were cultured under a 24-h photoperiod, and temperatures were maintained at 28 ± 2°C. After 4 to 6 weeks, plants regenerated from calli were transferred into glass jars (Sigma) containing 100 ml MS basal semi-solid medium and 3% sucrose until they fully developed roots, then were transplanted to sterile soil in a greenhouse.

Transgenic rice plants were induced with SA at pot or culture stage. In the pot treatment, rice plants were subjected to flooding in 5 mM SA for 1 day when the distance of the flag leaf auricle (of the primary tiller) to that of the next leaf was about 5 cm. Then anthers were inoculated in CIM medium without SA. Culture treatment involved adding 0.1 mM SA to the CIM medium. At least 100 anthers of each transgenic line were collected for experiments and all treatments were tested in a completely randomized design.

### PCR assay and DNA blot analysis

Genomic DNA was extracted from regenerated calli or plantlets of transgenic plants using a kit (Genemark, Tainan, Taiwan). Excision of *COKC* in transgenic plants was analyzed by PCR with three oligonucleotide primers: CF (5′-CGTTCAGTGCTGGTGGTCGT-3′), JR (5′-CTACAGCTCTTTTTGCAACTTTATC -3′) and DR (5′-CTTCTGCAGACTCCGGCGTG-3′) as described (Tai et al. 2011). The amplification protocol comprised 30 cycles of 1 min at 94°C, 2 min at 55°C, and 2 min at 72°C, and was performed in a T-gradient Thermocycler (Biometra, Göttingen, Germany).

The flanking sequences of the T-DNA or *COKC* integration sites in transgenic plants were determined by use of arbitrary degenerate primers and thermal assymeric interlaced PCR (TAIL-PCR) as described (Liu et al. 1995; Sha et al. 2004), with modification: primary TAIL-PCR involved approximately 150 ng of rice genomic DNA. The flanking sequences were amplified with the following oligonucleotide primers: TLnew4 (5′-GGTCAAGACCAATGTGGAGC-3′), TLnew3 (5′-GATTGTGTACGCCCGACAG-3′) and TLnew2 (5′-GGATTTTAGTACTGGATTTTGG-3′) for T-DNA and 3–1 (5′-GTGTGCTCCAGATTTATATGG-3′), 3–2 (5′-GATTTCGACTTTAACCCGACCGGA-3′) and 3–3 (5′-CGTTTTCGTTACCGGTATATCCCG-3′) for the 3′ end of *COKC*.

Regeneration plants from SA-induced anther culture were analyzed for homogeneity by PCR-dependent genotyping. The specific primers for flanking sequence and *COKC* are shown in Table 1. The thermal cycling program was 94°C for 5 min, followed by 35 cycles of 95°C for 1 min, 55°C for 30 seconds, 72°C for 1 min, and a dissociation cycle at 72°C for 1 min. For DNA blot, in brief, fresh leaves (2 g) or callus tissue (0.1 g) was frozen in liquid nitrogen and ground with use of a mortar and pestle. Nuclei were isolated and lysed by protease treatment, and genomic DNA was precipitated with ethanol and dissolved in TE buffer (10 mM Tris–HCl, 1 mM EDTA; pH 8.0). About 10 μg of each DNA was digested with the appropriate restriction enzyme under the conditions specified by the suppliers and fractionated on 0.8% agarose gels (in 1 × TAE) overnight at 1 V/cm. Southern blot analysis was performed as described (Charng and Pfitzner 1994).

**Table 1:** **Genomic sequences flanking** ***COKC*** **insertion in transgenic rice plants**

Line	Chromosome/BACs	Insertion position (bp)	GenBank accession no.	Identities	Primers	
					***COKC*** specific	Site specific
K19-H3	8/OSJNBa0095C12	46381	AY360385	231/231 (100%)	GGTATTTCTTACATGGGCTG	GCCTTTTGACATGTAGCG
						GTCATACATTCTGATCATGAG
K19-C2	2/OSJNBa0091C16	141691	AP005820	198/200 (99%)	GGTATTTCTTACATGGGCTG	GCCGGTGGAGTGTCCTCCG
						GGTCGGCCTTGTTCGAGCG
K19-H5	10/OSJNBa0094J09	56754	AC078839	89/89 (100%)	GGTATTTCTTACATGGGCTG	GCCATAGCAAAGATCAGATC
						GAAATCTATTGCGTGCCAG
K17-H3	7/P0045F02	30502	AP004268	344/344 (100%)	GGTATTTCTTACATGGGCTG	CATAAGTTGTAGCAAAGCATAC
						ATTCATTTCATCTCTCAATTA
K20-H6	1/OSJNOa264G09	14881	AP008219	256/256 (100%)	GGTATTTCTTACATGGGCTG	GCTGAAGACATTCAGAGGGAG
						CTGCAGAAAATAAGCATATT
K24-C2	12/OSJNBa0037B01	58801	AL928751	155/155 (100%)	GGTATTTCTTACATGGGCTG	CTGATGAGCAAGAGAAAAAG
						CCGCCTCCTGGTCAGCTC

Primers for determining the *COKC* homogeneity of each insertion line are indicated.

## Results

### Inducible transposon construct and generation of starter lines

**Figure 1:**
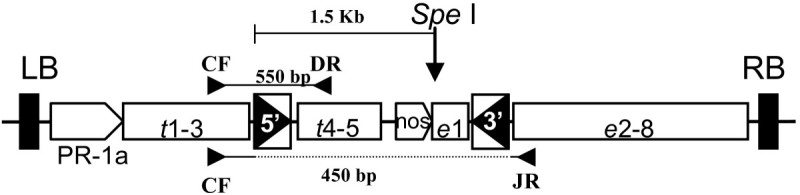
**Construction of the one-time inducible transposon,** ***COKC*** **.** The 5′ end was constructed in the third intron of the transposase gene, which was downstream of the PR-1a promoter. The 3′ end was constructed in the first intron of the selectable marker, modified *epsps* gene, involved in glyphosate resistance. Excision of *COKC* leads to a truncated transposase and marker genes and consequently a marker-off and defective *COKC. t*, transposase gene of *Ac* transposon (numbers indicate its corresponding 5 exons); *e*, modified rice *epsps* gene (numbers indicate its corresponding 8 exons). Restriction enzyme for Southern blot and primers for identifying excision events and expected PCR products are shown. The primer sets (CF, DR and JR) used to detect excision events are shown as a solid triangle. LB, left border; RB, right border; 5′ and 3′, *Ac* left and right-terminal inverted repeat; PR-1a, PR-1a inducible promoter; nos, nos promoter.

A schematic representation of the construct pCOKC used for transformation is shown in Figure 1. A one-time transposon was previously developed by locating one end of the transposon in the intron of the *Ac* transposase gene, under the control of the inducible PR-1a promoter, thus allowing the transposase gene to be expressed when the plants are treated with SA (Tai et al. 2011). This system was introduced into rice plants, and 34 transgenic lines containing a single copy of *COKC* were identified and the excision efficiencies analyzed. In total, 7 lines yielded SA-induced excision events and were chosen to check the efficacy of the system (Tai et al. 2011). Non-SA treated calli of these lines were analyzed first by PCR for spontaneous excision events. Five lines (K-17, K-19, K-20, K-21 and K-24) yielding no detectable signal for spontaneous excision were used as anther donor plants (data not shown).

### Induction of *COKC*

**Figure 2:**
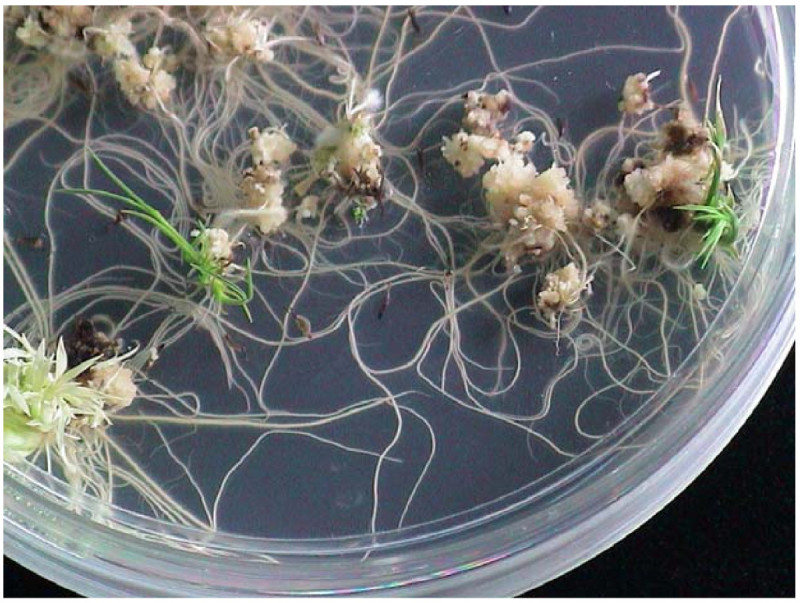
**Anther-cultured regenerating plantlets of transgenic rice harboring the** ***COKC*** **system, with floral tissues treated by salicylic acid (SA).**

The starter lines underwent anther culture and SA treatment to induce transposase activity and initiate mutagenesis. The siblings of each transgenic line were divided into 3 sets for induction experiments as described. As a control, one set underwent anther culture without SA treatment. The other transgenic lines of rice were induced at pot or culture stages and the SA concentration was set to 5 or 0.1 mM SA, respectively. For pot induction, higher SA-dose (10 or 50 mM) treatments produced yellowed flag leaves or decreased ability for callus regeneration (data not shown). Since it has been reported that low temperature shock would enhance the androgenic response in rice, all detached tillers were pre-treated at 10°C for 10 days (Ogawa et al. 1995; Rukmini et al. 2013). The excision events were determined by PCR and DNA blot analysis of regenerated calli or plantlets developed from anthers (Figure 2).

### Determination of transposition events

**Figure 3:**
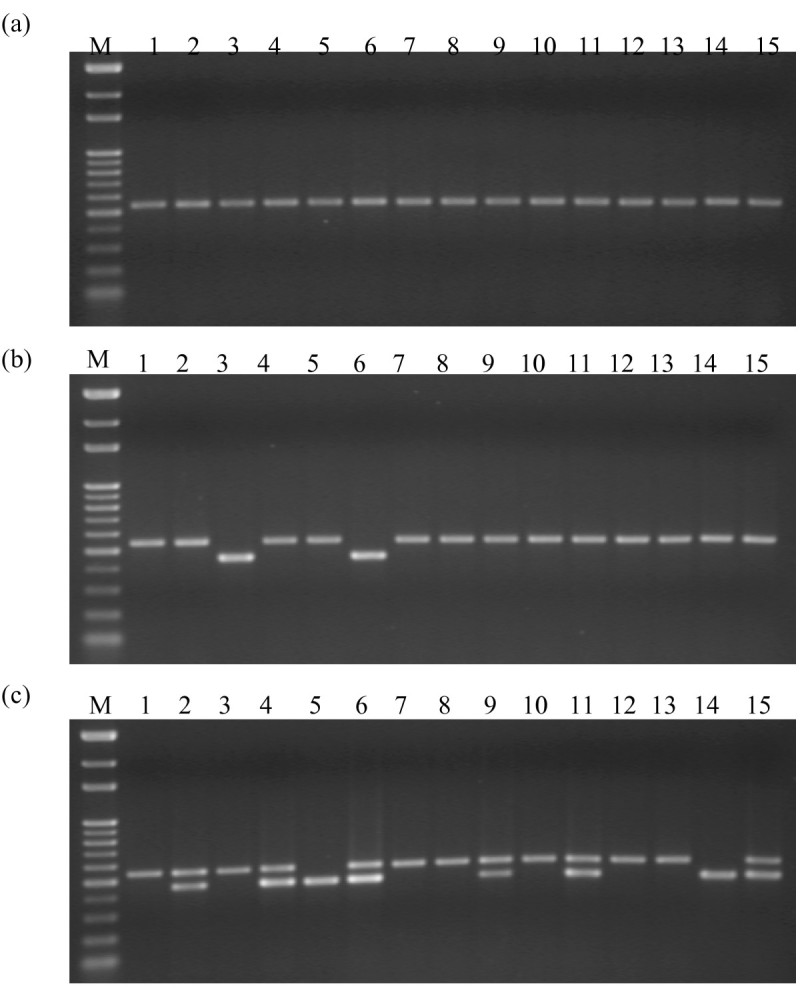
**PCR analysis to determine** ***COKC*** **excision events.** DNA was extracated from transgenic rice containing *COKC*; anthers were cultured normally **(a)** or were induced with SA at pot **(b)** or SA-containing medium **(c)**. Regenerated calli or shoots from each treatment were collected randomly for DNA for multiplex PCR reactions, which yielded 550- and 450-bp products, referred to as untransposed and transposed *COKC*, respectively. M, 100-bp ladders.

**Figure 4:**
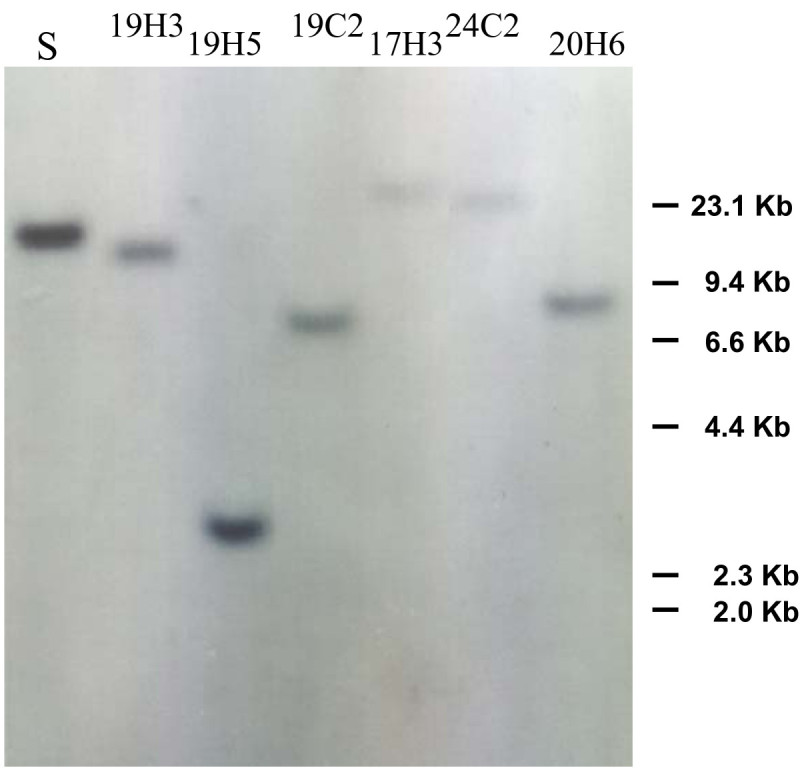
**DNA blot analysis of rice shoots regenerated from anther culture with SA induction.** S, K19 Start line.

After the induction process, the regenerated shoots from anthers were collected for DNA extraction. The excision events were determined by multiplex PCR analysis. The induced excision events and efficiency in each transformed line were determined by presence of the 450-bp and/or 550-bp fragment (Figure 3). At least 15 samples for each treatment from each starter line were collected for PCR analysis. Three primers were used to verify transposed vs. un-transposed *COKC* elements. With the primers CF and DR, a 550-bp PCR product was obtained with DNA from transgenic rice plants, which harbored the un-transposed *COKC* element (Figure 1). With the primers CF and JR, a 450-bp PCR product was obtained with DNA from transformed rice plants, indicating the excision of the *COKC* element. All shoots with control treatment yielded 550-bp fragments with primers CF and DR (Figure 3a) but no product with primers CF and JR. Therefore, the *COKC* elements were stable during anther culture without induction treatment. Among the eighty shoots regenerated from pot SA treatments, 76 yielded a 550-bp fragment and 4 a 450-bp fragment (Figure 3b, lanes 3 and 6; the other 2 not shown). The 4 rice plantlets were designated as K19-H3, K19-H5, K17-H3 and K20-H6, “H” indicating the pot treatment. These findings suggest that the excision event occurred with an efficiency of 5%. For culture treatment, 450-bp fragments were detected in 19 of 96 shoots regenerated with efficiency of 20% (Figure 3c). It was found that 17 shoots containing excision events yielded both a 550-bp and 450-bp fragment, hence it can be inferred that the transformed rice contained cells in which *COKC* had undergone excision and cells in which it had not. Therefore, the excision events occurred during the DH calli regeneration, and such somatic excision resulted in heterozygous lines. In contrast, the other 2 plantlets, K19-C2 and K24-C2 (Figure 3c, lanes 5 and 14), yielding a 450-bp fragment only, were determined as homozygous lines. For pot treatment, although the excision efficiencies were relative low, yielding a 450-bp fragment as the only product suggested that the excision events occurred in germinal cells. DNA gel-blot hybridization performed on the transposed lines involved use of the DNA fragment (containing the 5′ end, exon D and E of the transposase gene) as a probe to search for transposed *COKC* after plants underwent SA treatment. *Spe* I digestion of the genomic DNA resulted in a unique hybridizing band but larger than 1.5-Kb. A transposed COKC would yield new unique hybridizing band different from that of the starter line. Lines K19-H3, K19-C2 and K19-H5, regenerated from the SA-treated anther-cultured K19 starter line yielded new unique hybridizing bands (Figure 4). Thus, germinal transposition events occurred during the SA-treated anther culture and resulted in homogeneity of the transposed *COKC*.

### Determination of di-haploid (DH) mutants produced by *COKC*

**Figure 5:**
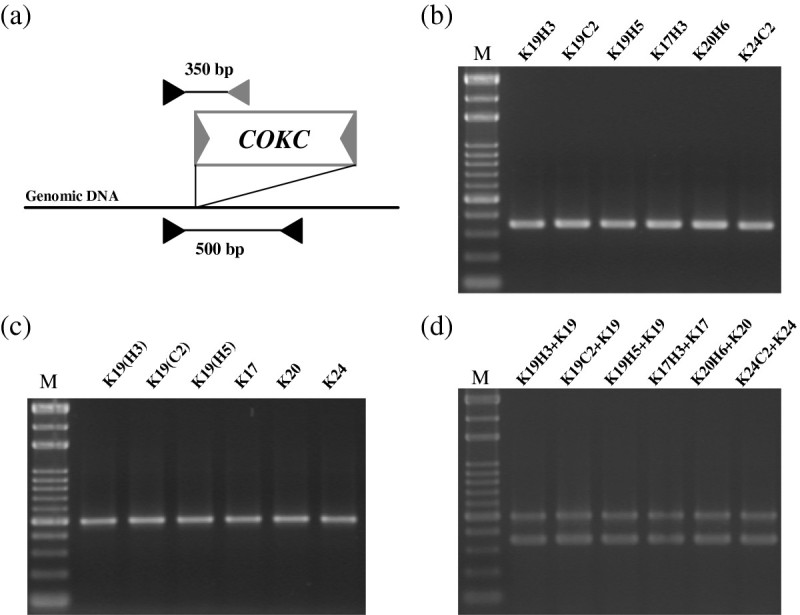
**PCR-dependent genotyping of homogeneity of the inserted** ***COKC*** **in 6 rice plants regenerated from SA-induced anther culture from their starter lines K19, K17, K20 and K24. (a)** Site-specific primers (solid triangle) were designed according the flanking sequences of each *COKC* insertion locus. These primers were combined with *COKC* specific primers (gray triangle) for multiplex PCR yielding a unique product of sizes of 500 or 350 bp for corresponding *COKC*-containing **(b)** or empty **(c)** sites, respectively. Both products were amplified only when mixing DNAs of each starter line and its regenerated progeny by anther culture **(d)**.

Anther culture may sometimes create haploids, which may have resulted in the unique products observed in Figures 3b (lanes 3 and 6) and 3c (lanes 3 and 14) and Figure 4. All 6 rice plants (K19-H3, K19-H5, K19-C2, K17-H3, K20-H6, and K24-C2) were further identified to be DH because of the ability to set seeds (data not shown). The flanking sequences for the *COKC* integration sites were analyzed by a thermal asymmetric interlaced (TAIL)–PCR-based approach (Liu et al. 1995; Charng et al. 2007). The sequences obtained (Table 1) were subjected to a BLAST search of the National Center for Biotechnology Information (NCBI) database. Site-specific primers were designed and used with *COKC*-end primers to verify the homogeneity of inserted *COKC* in the specific loci (Table 1). Specific primers were designed to yield 350-bp products with *COKC*-end plus site-specific primers and 500-bp products with site-specific primers alone (Figure 5a). To yield specific products and to eliminate cross contamination, these primers were combined for multiplex PCR to determine the expected DH mutants and their starter lines. The regenerated rice shoots were identified as bi-allelic with *COKC* integration by yielding only 350-bp products (Figure 5b). The 500-bp products were produced only in the starter lines with site-specific primers (Figure 5c). To rule out the possibility of any inhibition of each primer annealing to its specific target, we performed controlled PCR reactions by using a template DNA mixture of the starter and regeneration lines, which eventually yielded both 350- and 500-bp products (Figure 5d). Thus, SA-induced transposition in rice male gametes, followed by anther culture treatment, could yield independent DH mutants.

## Discussion

Anther culture is used specifically in plant systems to produce haploids and DHs through gametic embryogenesis and allows for a single-step development of complete homozygous lines from heterozygous parents. In a few wheat species, the efficiency to regenerate DH plantlets from anther culture can be up to 30% (Lantos et al. 2013). In general, in rice, the regeneration rate is more than 5 green plantlets/100 anthers. Thus, this technique has been further used with native transposon mutagenesis (Kikuchi et al. 2003; Dong et al. 2012). To promote transposition efficiency and control transposition specifically in germinal cells, we assessed the use of an inducible transposon, *COKC*, to create DH mutant rice plants. To rule out spontaneous transposition events without induction (Tai et al. 2011), we preliminarily identifed 5 starter lines. We used two induction methods and, with pot SA treatment, the germinal transposition efficiency was up to 5% from regeneration calli. All regenerated plantlets were identified as homozygous mutants (Table 1 and Figure 5), so the germinal transposition events were successfully induced. With culture treatment, the transposition efficiency was up to 20%. Yet, many regenerated plantlets yielded untransposed *COKC* signals (Figure 3c), so the transposition events occurred during calli regeneration, which resulted in heterogeneity of the transposed *COKC*. Considering that rice anther culture yields a 5% regeneration rate, our results indicate that incubating 2,000 anthers from starter lines containing *COKC* can yield approximately 100 independent regenerated rice plantlets and 6 homozygous mutants. Since a high regeneration rate (up to 25%) has been reported in a commercial rice line (Islam et al. 2004), our technique can reduce the number of starter anthers needed by 5-fold.

Since the rice genome has been fully sequenced, the flanking sequences of the transposed *COKC* were aligned to monitor whether any desired gene was tagged. In the transposed line K24-C2, *COKC* inserted in the hypothetical gene, whose translated product is similar to flavonoid 4′–O-methyltransferase. Yet we observed no phenotypic alteration in this or the other 5 adult rice mutants (data not shown).

Although we have demonstrated that *COKC* can create DH rice mutants, the use of inducible transposon mutagenesis for other plant species needs further investigation. Plant species with high anther-culture efficiencies, such as Solanaceae and Cruciferae, are a prerequisite for application of germinal transposition mutagenesis. For plant species whose genomes have been completely sequenced and most genes annotated (or are in progress; e.g., papaya), a mutant with the tagged loci, which identified by flanking sequence alignment and matched the annotated gene, can be used to observe the mutant phenotype directly. For plants without complete genome sequencing, the flanking-sequence information of the tagged loci is needed to confirm the homozygous state, which is helpful for phenotype-driven genetic screening.

## Conclusions

*COKC* has been designed for one-time induction, so the possibility of subsequent transposition of the transposed *COKC* by endogeneous stimuli can be ruled out. Yet, the transposition efficiency, specifically in germinal cells, needs to be improved. Additional developments for plant-gene tagging systems with anther culture could replace the promoter with a mid- to late-uninucleate microspore specific one so that the transposition is stable after integration. All of these promoters, combined with the one-time inducible transposon concept, will allow us to develop more efficient transposon systems for plant functional genomic studies.

## Authors’ original submitted files for images

Below are the links to the authors’ original submitted files for images. Authors’ original file for figure 1 Authors’ original file for figure 2 Authors’ original file for figure 3 Authors’ original file for figure 4 Authors’ original file for figure 5

## Abbreviations

TE: Transposable element
SA: Salicylic acid
DH: Di-haploid.
