# Supplementary figures and images for: Application of an inducible transposon with anther culture in generation of di-haploid homologous mutants
Source: Bot Stud. 2014 Feb 24;55:27. doi: 10.1186/1999-3110-55-27 (PMC5432829; doi:10.1186/1999-3110-55-27)

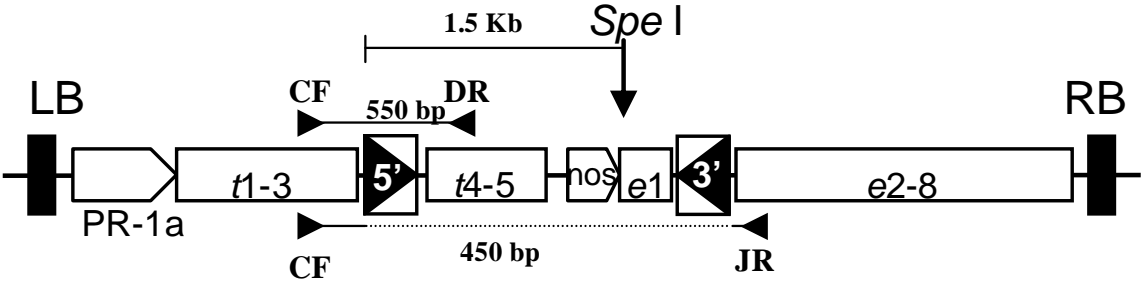

Supplement: Supplementary file 1 — Authors’ original file for figure 1 [file 40529_2013_78_MOESM1_ESM.pdf]

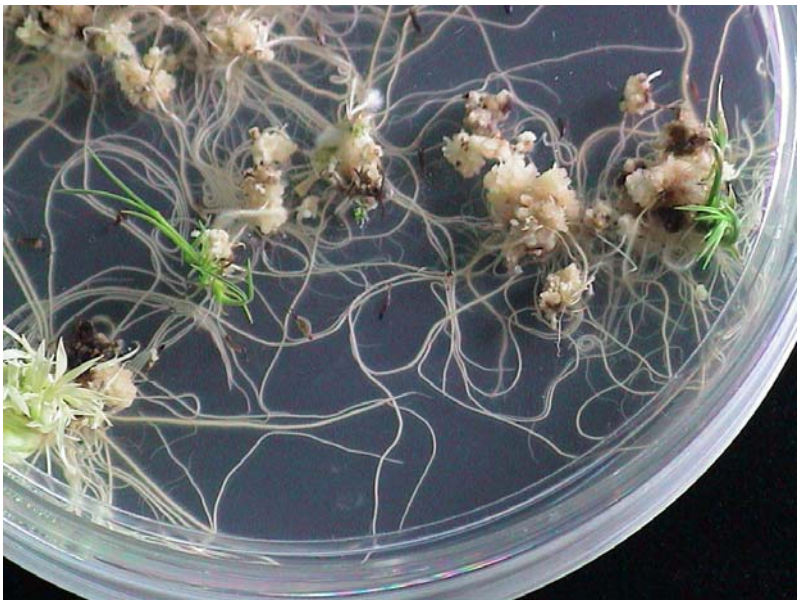

Supplement: Supplementary file 2 — Authors’ original file for figure 2 [file 40529_2013_78_MOESM2_ESM.pdf]

(a)

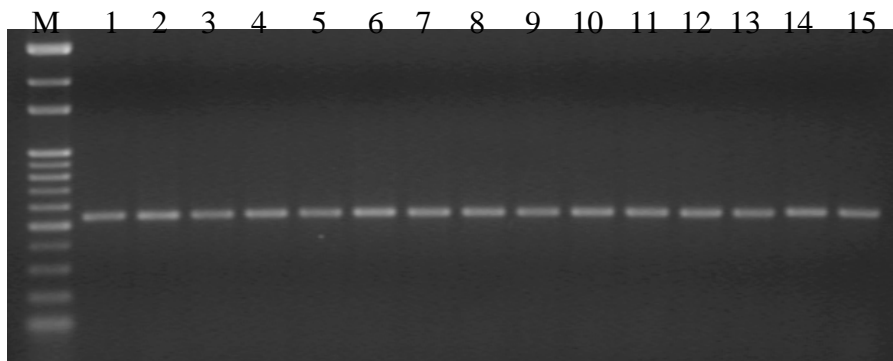

(b)

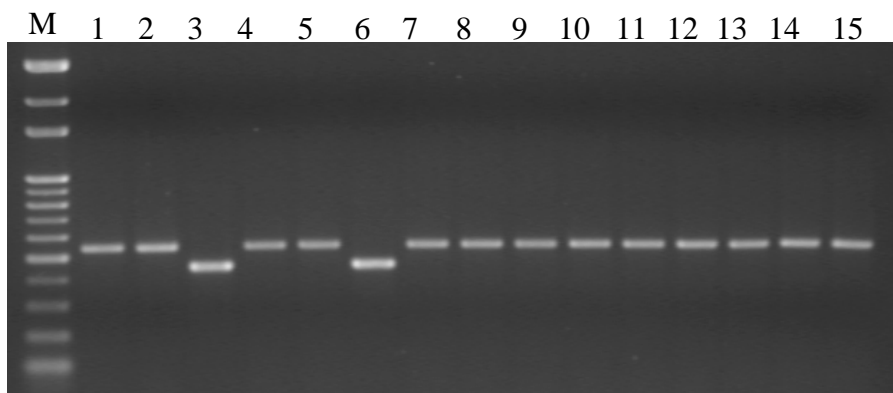

(c)

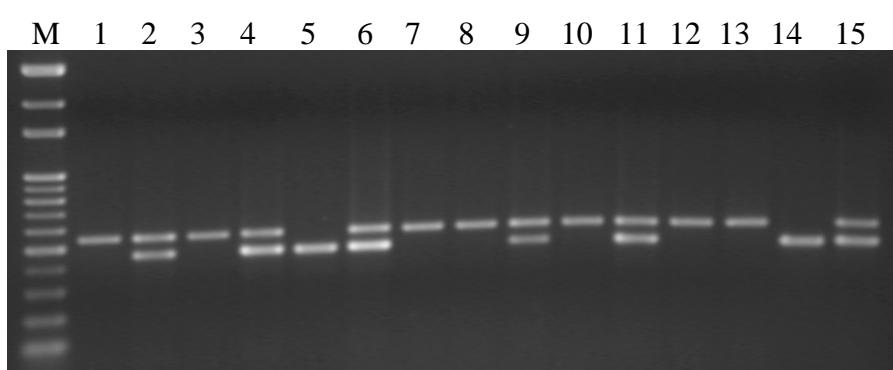

Supplement: Supplementary file 3 — Authors’ original file for figure 3 [file 40529_2013_78_MOESM3_ESM.pdf]

S    19H3   19H5   19C2   17H3   24C2   20H6

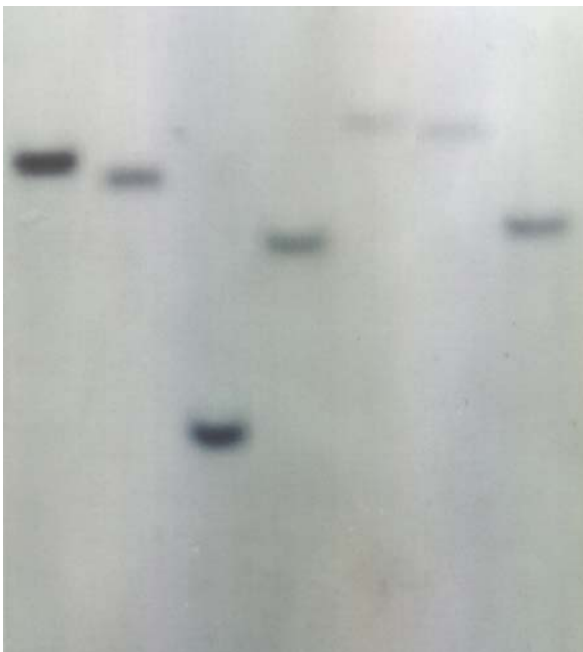

— 23.1 Kb

— 9.4 Kb

— 6.6 Kb

— 4.4 Kb

— 2.3 Kb

— 2.0 Kb

Supplement: Supplementary file 4 — Authors’ original file for figure 4 [file 40529_2013_78_MOESM4_ESM.pdf]

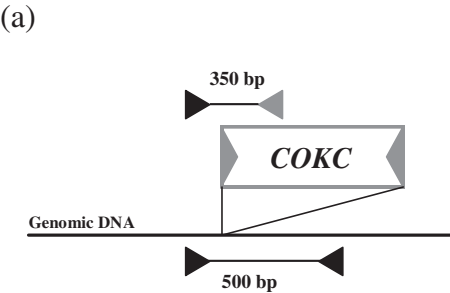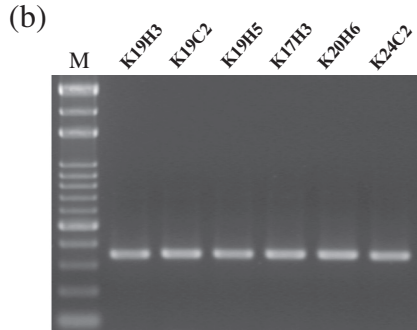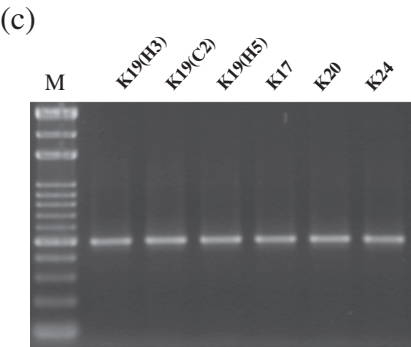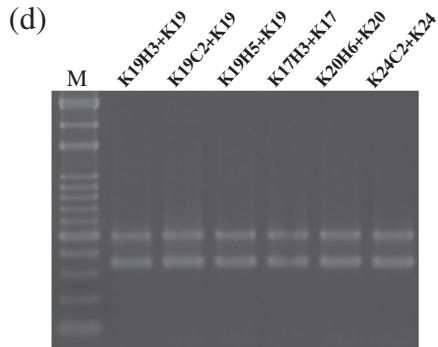

Supplement: Supplementary file 5 — Authors’ original file for figure 5 [file 40529_2013_78_MOESM5_ESM.pdf]
